# Current and Novel Reviews in Sports Nutrition

**DOI:** 10.3390/nu13082549

**Published:** 2021-07-26

**Authors:** David C. Nieman

**Affiliations:** Human Performance Laboratory, North Carolina Research Campus, Department of Biology, Appalachian State University, Kannapolis, NC 28081, USA; niemandc@appstate.edu; Tel.: +1-828-773-0056

Sports nutrition is a rapidly expanding area of scientific investigation and is being driven by high interest from both the academic community and the exercising public [1]. Research into the discipline of sports nutrition is challenging. The interaction of exercise and nutrition is both complex and compelling, with an endless array of potential sports nutrition products, pathways, and hypotheses to be tested. Some proposed sports nutrition strategies and products are innovative while others are based on fanciful conjecture [2]. 

Research designs for sports nutrition-based studies need to adhere to the highest quality standards to determine efficacy [1,3]. Nutrition dosing regimens are always challenging and, unfortunately, these are often based more on educated guesses and marketing issues than careful science. Many sports nutrition products have blends of macro- and micro-nutrients and phytochemicals that have clinical backing for some of the individual ingredients but not the entire mixture. Few sports nutrition products have been tested for stability, absorption, disposition, metabolism, and excretion. 

A “food first” approach for athletes is recommended by most sports nutrition professionals. The problem for some athletes, however, is that they are resistant to adopting dietary patterns that are consistent with published guidelines. For many people that exercise, a healthy dietary pattern is sufficient to supply the nutrients needed to support a healthy response to increased exercise levels [4]. High-level athletes may need extra help beyond the food supply to meet the nutrient demands of stressful exercise workloads, but this is still being debated [1]. Nonetheless, adaptations within a healthy dietary pattern may support both performance and health for even the athletes with the most demanding training programs. For example, recent studies support that fruit and water consumption can complement or even take the place of commercial sports beverages for those exercising intensely for long periods of time [5,6,7]. 

Sports performance can be measured in many different ways, and nutritional interventions are often evaluated as essential when performance is improved. Some sports nutrition products are targeted for outcomes that are not easily felt by the athlete, including lowered inflammation, immunosuppression, and oxidative stress, and enhanced metabolic recovery [8,9]. This type of benefit is not easily conveyed to the athletes or coach, or in particular, the exercising public. 

Advances in measurement technologies are allowing hundreds of metabolites, proteins, lipids, and genes to be measured at one time, improving the capacity to provide accurate and practical guidelines for consumers. Nutrition and exercise have huge effects on nearly every system of the body, both acutely and chronically, and a human systems biology approach, although expensive, is needed to advance scientific understanding [10].

For this Special Issue, research leaders in sports nutrition were approached and invited to submit current reviews in their areas of expertise [11,12,13,14,15,16,17,18,19,20,21]. The topics are novel and wide-ranging, and include updates and insights on protein [11,12], dietary patterns and nutritional interventions to support sleep, older athletes, and sports performance [13,14,15], pre-exercise nutrition [16], supplementation with betaine, iron, and creatine [17,18,19], and sports nutrition research methodologies for body composition and muscle glycogen analysis [20,21]. A major emphasis in all of the papers was a focus on strengths and weaknesses for various sports nutrition strategies, and insights for future research.

Kerksick et al. [11] defined the role that proper doses of plant proteins can have in supporting health, the environment, and exercise training adaptations. The systematic review and meta-analysis by Chapman et al. [12] concluded that protein supplementation improves strength and muscle mass during intensive and long-term training. Nutritional interventions, such as supplementation with tart cherry juice, kiwifruit, 20–40 grams of protein rich in tryptophan, and glycine late in the day were recommended as useful sleep-enhancement strategies for athletes in the narrative review by Gratwicke et al. [13]. Strasser et al. [14] focused on nutritional guidelines for older adults, including adequate energy and protein intake for countering losses in bone and muscle mass. 

Five popular dietary patterns, including vegetarian diets, high-fat ketogenic diets, intermittent fasting diets, gluten-free diet, and low fermentable oligosaccharides, disaccharides, monosaccharides and polyols (FODMAP) diets were reviewed by Devrim-Lanpir [15]. This comprehensive review summarized both the beneficial and detrimental features of each of these diets on athletic performance. Pre-exercise nutrition is an important and current issue in sports nutrition, and Rothschild et al. [16] provided a detailed explanation of how the availability of endogenous and exogenous carbohydrate, fat, and protein before and during exercise can influence adaptations to endurance exercise. 

Betaine (trimethylglycine) can be made in the body from choline or consumed in the diet from wheat bran and germ, spinach, and beets. Betaine is a methyl donor and helps regulate intracellular fluid concentrations and cell volume. Willingham et al. [17] argued that human clinical trials are needed to confirm whether or not betaine supplementation can improve safety and exercise performance in heat, as supported by animal studies.

Low carbohydrate and energy intake can negatively influence iron status in athletes and is often mediated by hepcidin expression. The comprehensive narrative review by McKay et al. [18] recommended that athletes shorten the duration of low carbohydrate training periods to minimize potential effects on hepcidin and iron regulation. Creatine is one of the most popular sports nutrition supplements on the market, and Arzi et al. [19] presented emerging evidence that creatine supplements may play a role in countering exercise-induced oxidative stress.

Kasper et al. [20] provided an excellent overview of body composition testing methodologies. This research group concluded that properly conducted skinfold measurements provide useful data and may be preferred over other methods because it is simple, low-cost, least affected by lifestyle confounders, and good for the long-term tracking of athletes. The measurement of muscle glycogen is important in sports nutrition studies, and Bone et al. [21] cautioned that high-frequency ultrasound technology for estimating muscle glycogen content needs further development.

This Special Issue on sports nutrition provided current updates in many core areas, with insights from leading experts for future research. Hopefully scientific understanding will be advanced as these ideas are converted into novel research designs and discoveries.

## Data Availability

Not applicable.
